# Boosting Multi-Vehicle Tracking with a Joint Object Detection and Viewpoint Estimation Sensor

**DOI:** 10.3390/s19194062

**Published:** 2019-09-20

**Authors:** Roberto J. López-Sastre, Carlos Herranz-Perdiguero, Ricardo Guerrero-Gómez-Olmedo, Daniel Oñoro-Rubio, Saturnino Maldonado-Bascón

**Affiliations:** 1 GRAM, Department of Signal Theory and Communications, University of Alcalá, 28805 Alcalá de Henares, Spain; c.herranz@edu.uah.es (C.H.-P.); saturnino.maldonado@uah.es (S.M.-B.); 2 BBVA Next Technologies, 28050 Madrid, Spain; ricardo.guerrero.gomez-olmedo.next@bbva.com; 3 NEC Labs Europe, Kurfürsten-Anlage 36, 69115 Heidelberg, Germany; daniel.onoro@neclab.eu

**Keywords:** traffic monitoring sensor, vehicle tracking, vehicle detection, tracking by detection, viewpoint estimation, smart city

## Abstract

In this work, we address the problem of multi-vehicle detection and tracking for traffic monitoring applications. We preset a novel intelligent visual sensor for tracking-by-detection with simultaneous pose estimation. Essentially, we adapt an Extended Kalman Filter (EKF) to work not only with the detections of the vehicles but also with their estimated coarse viewpoints, directly obtained with the vision sensor. We show that enhancing the tracking with observations of the vehicle pose, results in a better estimation of the vehicles trajectories. For the simultaneous object detection and viewpoint estimation task, we present and evaluate two independent solutions. One is based on a fast GPU implementation of a Histogram of Oriented Gradients (HOG) detector with Support Vector Machines (SVMs). For the second, we adequately modify and train the Faster R-CNN deep learning model, in order to recover from it not only the object localization but also an estimation of its pose. Finally, we publicly release a challenging dataset, the GRAM Road Traffic Monitoring (GRAM-RTM), which has been especially designed for evaluating multi-vehicle tracking approaches within the context of traffic monitoring applications. It comprises more than 700 unique vehicles annotated across more than 40.300 frames of three videos. We expect the GRAM-RTM becomes a benchmark in vehicle detection and tracking, providing the computer vision and intelligent transportation systems communities with a standard set of images, annotations and evaluation procedures for multi-vehicle tracking. We present a thorough experimental evaluation of our approaches with the GRAM-RTM, which will be useful for establishing further comparisons. The results obtained confirm that the simultaneous integration of vehicle localizations and pose estimations as observations in an EKF, improves the tracking results.

## 1. Introduction

Many intelligent transportation systems need a robust and fast sensor for detecting and tracking multiple vehicles. Some examples are vehicle speed estimation solutions [1,2], illegal parking detection modules [3], traffic estate estimation systems [4,5,6] or vehicle detection in aerial images for surveillance [7,8].

In this work we introduce a new intelligent vision based sensor able to perform multi-vehicle tracking by a joint object detection and coarse viewpoint estimation. Typically, in a multi-vehicle tracking-by-detection approach, a fundamental part of the system pipeline is the object detection step. However, with this paper we want to show that it is also crucial to incorporate to the tracking model the observations for the viewpoints of the vehicles, that is, the pose of the vehicle with respect to the camera. Can we *recover* this information jointly during the detection step in a fast way? How can we efficiently integrate these pose observations into the tracking model? These are some of the questions we want to answer with this work.

Category-level object detection in images and videos has been a very popular research theme over the last years (e.g., References [9,10,11,12,13,14,15,16,17,18]), where the objective of all these works has been to predict the bounding boxes in the images where the objects of interest appear.

**Figure 1:**
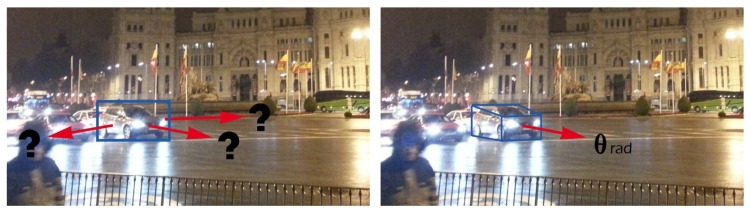
We humans are able not only to detect an object but also to estimate its viewpoint or pose. Furthermore, we are able to use this *semantic* information to estimate a likely direction for the movement of the object of interest. For instance, if a car is observed under a frontal orientation, we will predict that it will move towards the camera position. Reprinted by permission from Springer Nature: Springer, Natural and Artificial Computation in Engineering and Medical Application, (Vehicle Tracking by Simultaneous Detection and Viewpoint Estimation, Guerrero-Gomez-Olmedo, R. et al.), 2013.

Some recent works also propose to deal with the problem of estimating the viewpoint of the objects (e.g., References [19,20,21,22,23,24,25,26,27]). It is, therefore, a more difficult problem, because in addition to localizing the object, the approach has to estimate a point of view for it. From the different analysis performed, it seems that the best way to tackle this problem consists in decoupling the localization and the pose estimation tasks [27], so that the object detection performance does not degrade. In any case, we do believe that this technology can be beneficial for a tracking model. For instance, if we humans look at the car shown in Figure 1, we are able to infer its pose and consequently to predict a *likely* direction for its movement. Therefore, we propose to enhance the tracking algorithm with observations of the vehicle position and viewpoint, which results in a better estimation of the vehicles trajectories, as our experimental evaluation shows.

To analyze the effect of the integration of the viewpoint observations into the tracking model, in this work we propose a novel approach for multi-vehicle tracking-by-detection with simultaneous coarse viewpoint estimation. Our contributions are threefold:

- First, we develop the multi-vehicle tracking approach with a new design for an Extended Kalman Filter (EKF) which is able to simultaneously integrate into the motion model both the position and the viewpoint observations of the objects captured by our sensing solution.
- Second, we introduce two solutions for the simultaneous object detection and pose estimation problem. One consists in a GPU-based implementation of a Histogram of Oriented Gradients (HOG) detector with Support Vector Machines (SVMs) to estimate the viewpoints. For the other, we modify and train the Faster R-CNN deep learning model [16], in order to recover from it not only the object localization but also an estimation of its pose.
- And third, we publicly release a thorough experimental evaluation on the challenging dataset for multi-vehicle tracking and detection, the GRAM Road Traffic Monitoring (GRAM-RTM) database. It has been especially designed for evaluating multi-vehicle tracking approaches within the context of traffic monitoring applications. It comprises more than 700 unique vehicles annotated across more than 40.300 frames of three video sequences. We expect the GRAM-RTM becomes a novel benchmark in vehicle detection and tracking, providing the computer vision and intelligent transportation systems community with a standard set of images, annotations and evaluation procedures for multi-vehicle tracking.

We experimentally validate in the GRAM-RTM dataset our EKF based tracking system, using our approaches for simultaneous localization and pose estimation of vehicles. The results obtained confirm that the simultaneous integration of vehicle localizations and pose estimations as observations in an EKF, improves the tracking results.

A preliminary version of this work was published in Reference [28]. For this journal paper: (a) we have extended the technical and theoretical analysis of the multi-vehicle tracking solution; (b) we also incorporate a novel model for the object detection and pose estimation, that is, the one based on the Faster R-CNN; (c) we have also made a significant extension of the experimental validation; and (d) we detail and publicly release a revised version of the GRAM-RTM dataset.

The rest of this paper is organized as follows. Section 2 provides a review of related work in multi-object tracking, detection and pose estimation. In Section 3 we introduce a detailed description of the proposed tracking solution. In Section 4 GRAM-RTM Database is described along with the metrics proposed to evaluate the performance of the different algorithms. Finally, Section 5 describes the experimental results and Section 6 provides our main conclusions.

## 2. Related Work

We can say that multi-target tracking (MTT) in videos is a well established line of research. An excellent survey of recent and traditional tracking algorithms can be found in Reference [29].

This MTT task is normally decomposed into two coupled problems: (i) state estimation; and (ii) data association. Different solutions for the state estimation and motion models have been proposed in the past for a variety of application scenarios (e.g., References [30,31]).

With respect to the data association problem, much of the existing literature on MTT can be categorized into three main groups. These include global nearest neighbor (GNN) (e.g., Reference [32]), joint probabilistic data association filters [33] and Multiple Hypothesis Tracking (MHT) (e.g., Reference [34]). The MHT technique is able to maintain many possible data association hypotheses and propagate the corresponding target state estimates for each hypothesis. This kind of algorithm allows extremely complex MTT applications such as vehicle tracking from aerial images at high altitude and with a very low frame-rate [35].

Several tracking algorithms have been proposed. There are approaches, such as that in Reference [36], which deal with the complexity of real-time tracking in uncontrolled environments with unexpected lighting changes or even low contrast. We find also the color based Mean-Shift [37] and the Cam-Shift [38] classic techniques. However, they are not suitable for monitoring a complex traffic urban scene, typically crowded with vehicles and pedestrians moving and where the cameras change too. Furthermore, in these environments, works based mainly on movement detection, such as that in References [39] or [40], could not be satisfactorily employed either.

Probably, the best option for monitoring these scenarios are the tracking-by-detection based approaches [6,41,42,43,44]. However, a common problem of most of them is that the bounding boxes are not adequate to constrain the object motion sufficiently. This complicates the estimation of a robust trajectory. On the other hand, following the tracking-by-detection philosophy one is able to work in complex scenes and to provide an automatic reinitialization by a continuous application of an object detector sensor.

In order to overcome the limitations of the tracking-by-detection methods, we do integrate our simultaneous viewpoint estimation and object detection module in a tracking-by-detection architecture with an EKF.

Tracking using viewpoint estimation solutions is a much less explored area. Some tracking approaches use a 3D model of a particular target object in order to estimate its precise pose [45,46]. However, it is not feasible to use these techniques in a *real* urban scenario, where instead of a particular object, the system has to track *object categories*. Moreover, these techniques are not designed to deal with multiple objects present in the scene.

In our approach, we propose to integrate into our multi-vehicle tracking-by-detection model, a coarse viewpoint estimation module. This way, we can integrate into the EKF not only the object localization but also the observations of its viewpoint, given by our intelligent sensing solution, with the aim of enhancing the vehicle’s motion prediction.

In Reference [47], a vehicle tracking model using particle filters and rough viewpoint estimations is proposed. The incorporation of the viewpoint information is made by varying the horizontal variance in the set of particles when a vehicle is recognized as a side or frontal view (e.g., for a side view the horizontal variance should increase). In contrast, our approach is able to integrate the pose estimation of the vehicle *directly* into the motion model. That is, our observations for the pose directly modify the orientation of the vehicle in the model. Furthermore, in our formulation a continuous viewpoint estimation, not coarse, can in principle be integrated, providing a more precise pose observation for the motion model.

Table 1 shows a comparison where we clarify the main contributions of our solution with respect to state-of-the-art solutions for multi-vehicle tracking-by-detection solutions.

To deal with the problem of estimating the pose of vehicles and other object categories (e.g., References [23,48,49]) has been also recently addressed. For instance, discriminative models are introduced in References [48] and [49], which are based on a mixture of HOG templates for a joint vehicle localization and pose estimation. In Reference [23], the Deformable Part Model (DPM) [11] is extended to a 3D object model. Their solution consists of multiple parts modeled in 3D and a continuous appearance model. However, these previous models are not suitable to perform a fast detection of the vehicles, to be used in a tracking solution, for instance.

Our first approach builds on the state-of-the-art fast object detection solution of Reference [15] and the pose estimation model in Reference [19]. Specifically, we propose to learn a system for simultaneous detection and viewpoint estimation, following a similar learning strategy to the one introduced in Reference [19], which uses a DPM detector but for training the fast groundHOG detector [15] to simultaneously detect the car and predict a coarse viewpoint estimation. Our second solution leverages the object detection capacity and speed of the Faster R-CNN [16]. We simply incorporate to the original Faster R-CNN architecture a final softmax layer in order to achieve the pose estimation as a classification problem. These pose observations of these two solutions are further integrated into the dynamic motion model of an EKF, which is used to track the vehicles in the scene. Although there are some recent works that propose different solutions for the problem of real-time object detection (e.g., Reference [13]) and tracking (e.g., Reference [6]), our approach solves the coarse viewpoint estimation as well as the object localization problems, simultaneously and fast.

## 3. Tracking-By-Detection and Viewpoint Estimation

In this section we introduce a new approach to address the problem of multi-vehicle tracking via simultaneous detection and viewpoint estimation. Technically, ours is a tracking-by-detection approach which incorporates the observations for the pose of the objects into the motion model of an EKF. By adequately parameterizing the scene, an object localization can be accompanied by a viewpoint estimation for the vehicle, which is subsequently associated to an orientation for its movement (see Figure 1).

For the tracking system, we have chosen an EKF [50]. Although a simple motion model considering only the position and the speed of the vehicles using a Kalman Filter (KF) is enough in some cases, to enhance the tracking precision, we use the discrete and non-linear version of the KF, that is, the EKF, with the Ackermann steering model [51] (see Figure 3) for characterizing the vehicle’s non-holonomic motion. One of its main disadvantages is that, as the EKF is a Taylor’s linearized version of the KF, it quickly diverges if the process is not perfectly modeled or if we are not able to get measurements in a certain interval. We try to avoid these limitations using the *pose observations* from our intelligent vision sensor, simultaneously recovered from the detector, to estimate the orientation of the object movement. In order to track vehicles in crowded urban scenes, where occlusions and abrupt changes of trajectories are the main problems to deal with, our EKF approach results very convenient, as the experimental validation shows.

We start briefly describing the object detection and viewpoint estimation solutions in Section 3.1. Then, we offer a detailed description of how we integrate these observations into the object tracking pipeline (Section 3.2).

### 3.1. Simultaneous Vehicle Detection and Pose Estimation

The basis for a tracking-by-detection and viewpoint estimation approach are the object *detections*, which we define as follows,

(1) $${\bar{d}}_{t}^{\left(i\right)}={\left[x_{t}^{\left(i\right)},y_{t}^{\left(i\right)},\theta_{t}^{\left(i\right)}\right]}^{T}\;,$$

where, $\left(x_{t}^{\left(i\right)},y_{t}^{\left(i\right)}\right)$ encodes the 2D position of the object, that is, the localization and $\theta_{t}^{\left(i\right)}$ corresponds to the estimation of the viewpoint, for an object *i* at time stamp *t*. For the sake of clarity, we shall mostly omit the superscript *i* in the following. Note that our detections/observations of vehicles consist of not only the coordinates defining the localization of the objects but also their estimated poses, that is, $\theta_{t}^{\left(i\right)}$.

For solving this problem we propose two solutions. The first one is inspired in the pose estimation of Reference [19]. We here propose to learn a set of coarse viewpoint vehicle detectors, jointly trained for four particular discrete viewpoints: frontal, rear, left and right. Our work differs from Reference [19] in the type of detector used. Because one of our objectives is to perform a *fast* joint detection and pose estimation, we cannot directly follow Reference [19] using their modified DPM [11]. Instead we proceed to adapt the learning strategy of Reference [19] to train a GPU-based detector using HOG features, that is, the groundHOG [15].

The groundHOG detector relies on a high parallelization due to its implementation on GPU employing NVIDIA’s CUDA architecture. It follows a similar pipeline as the GPU approach presented in Reference [52], by computing gradients, histograms and SVM evaluations on GPU, while computing a multi-scale non-maximum-suppression step in CPU using the meanshift algorithm. In contrast to Reference [52], it is more loyal to the original CPU-based detector [10], for example, by padding the image with the color of its closest pixel from the original image instead of filling it with zeros. It includes some optimizations that realizes a significant speedup as compared to the original algorithm. It is also worth to mention that this approach has the ability to run multiple detectors in parallel, sharing the initial HOG feature computation. This results ideal for our purpose of pose estimation, because we can simultaneous run the four different coarse viewpoint estimators.

So, in this work, we introduce the following procedure to integrate viewpoint estimations directly into the groundHOG detector. Let us define the set of discrete viewpoints $\left\{\theta_{1},\theta_{2},\ldots ,\theta_{V}\right\}$, where *V* is the number of viewpoints. For our model, we use V=4, being the poses considered {frontal, rear, left, right}. We start learning a set of HOG templates, using linear SVMs as in Reference [10]. We learn one per discrete viewpoint $\theta_{v}$, called component HOG${}_{\theta_{v}}$. Each of these components, is refined using hard negatives. Because the objective is to provide a precise pose estimation, when training for a particular view (e.g., frontal), the negative examples may be extracted from images with the same object class but from the opposite viewpoint (e.g., rear), as it is detailed in Reference [19].

Once the set of HOG-based components {HOG${}_{\theta_{1}}$, HOG${}_{\theta_{2}}$, …, HOG${}_{\theta_{V}}$} has been learned, during the detection step we use groundHOG. In order to integrate all the outputs for the different viewpoints into a single detection response, we follow a bounding box-based non-maximum-suppression step on all the outputs of all the components HOG${}_{\theta_{v}}$. After applying the bounding box prediction for each component HOG${}_{\theta_{v}}$, we have a set of localizations and viewpoint estimations *D* in an image. Each detection is defined by a bounding box and an associated score. We sort the detections in *D* by their score and greedily choose the highest scoring ones while skipping detections with bounding boxes that are at least 50 percent covered by a bounding box of a previously selected detection.

**Figure 2:**
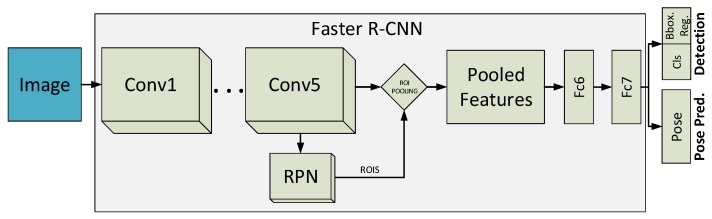
Simultaneous object detection and pose estimation solution for the Faster R-CNN architecture, which integrates the Region Proposal Network (RPN).

For our second approach, we propose a natural extension of the Faster R-CNN [16] for the problem of simultaneous object detection and pose estimation. Technically, we propose to add an extra output layer in order to predict the viewpoint of the object (See Figure 2). To understand the extension proposed, we proceed with a description of the original Faster R-CNN pipeline.

The Faster R-CNN consists of three stages. The first stage is performed by the convolutional layers. An input image passes through the convolutional part, to be transformed into a deep feature map. The second stage is represented by the Region Proposal Network (RPN), which serves as an attention mechanism during learning. Technically, it is a fully convolutional (sub)network, which takes an image feature map as input and outputs a set of rectangular object proposals, with their corresponding objectness scores. To go into details, RPN takes the feature map obtained from the last convolutional layer (e.g., convolution 5 in a VGG16-based architecture) and adds a new convolutional layer which is in charge of learning to generate regions of interest (ROIs). In the third stage, these ROIs are used for pooling those features that are passed to the last two fully-connected (FC) layers. Finally, the responses coming from the last FC layer are used by the model: (1) to classify the ROIs into background or object; and (2) to perform a final bounding box regression for a fine-grained localization of the object.

In order to incorporate the capability of pose estimation into the Faster R-CNN pipeline, guaranteeing a minimal intervention in the model architecture, we propose to incorporate an additional output softmax layer, connected to the last FC layer as well. The objective of this layer is to cast a prediction for the viewpoint and to measure the loss for this task, propagating the appropriate gradients to the rest of the network during learning.

For training this deep model, we minimize the following loss function,

(2) $$L\left(W,S\right)=\lambda_{c}L_{c}\left(W,S\right)+\lambda_{d}L_{d}\left(W,S\right)+\lambda_{p}L_{p}\left(W,S\right)\;,$$

where *W* encodes the parameters of the deep model, and *S* represents the training set of samples in the batch. $L_{c}$ and $L_{d}$ are the original classification and object detection losses detailed in Reference [16], with their corresponding weights $\lambda_{c}$ and $\lambda_{d}$, respectively. Note we simply add an extra loss for the pose estimation problem, that is, $L_{p}$, which is a standard cross entropy loss, as $L_{c}$, for the pose estimation problem. Again, as for the previous model, we train the network to classify the samples using four viewpoints, {frontal, rear, left, right}. We give the same weight to each task, that is, $\lambda_{c}=\lambda_{d}=\lambda_{p}=1$.

This way, the object detection step is able to feed the tracking motion model with object detections like ${\bar{d}}_{t}$, which incorporate the localization of the object (center of the bounding box) and the estimated pose. For the latter, the index of the winner component of the non-maximum-suppression HOG${}_{\theta_{v}}^{*}$ has to be converted to the corresponding objects’ trajectory orientation. In our model, this is done by parameterizing the viewpoint estimations in the scene. This way, an object localization can be associated to a viewpoint estimation, which is subsequently related to an orientation in the scene which describes the movement of the object.

### 3.2. Multi-Vehicle Tracking

**Figure 3:**
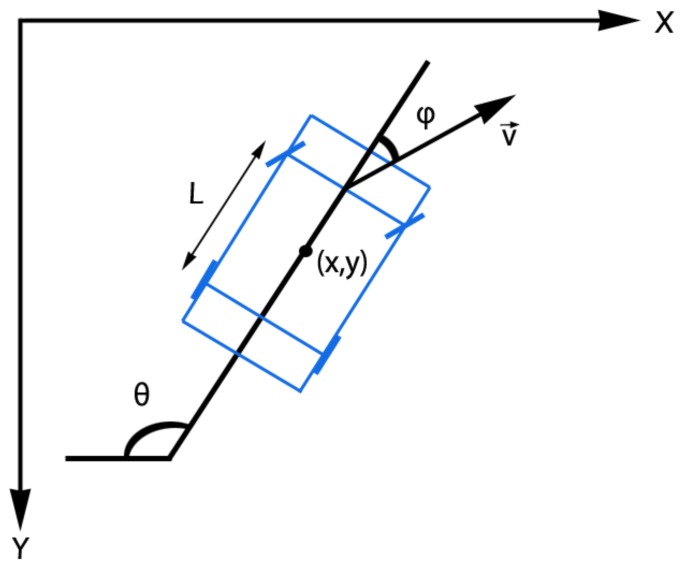
Ackermann’s Steering model [51]. Reprinted by permission from Springer Nature: Springer, Natural and Artificial Computation in Engineering and Medical Application, (Vehicle Tracking by Simultaneous Detection and Viewpoint Estimation, Guerrero-Gomez-Olmedo, R. et al., 2013.)

We define a measurement vector ${\bar{z}}_{t}\in R^{3}$ as ${\bar{z}}_{t}={\left[x_{t},y_{t},\theta_{t}\right]}^{T}$ and we assume that the tracking process has a state vector ${\bar{x}}_{t}\in R^{6}$ as ${\bar{x}}_{t}={\left[x_{t},y_{t},\theta_{t},v_{t},\phi_{t},a_{t}\right]}^{T}$, where: $x_{t}$ and $y_{t}$ encode the position of the object in the image (i.e., the center of the bounding box), $\theta_{t}$ defines the orientation of the movement, $\phi_{t}$ is the steering angle and $v_{t}$ and $a_{t}$ are the linear speed and the tangential acceleration, respectively. This formulation corresponds to the Ackermann’s steering model for cars [51]. See Figure 3 for a graphical representation of this dynamic model. Note that for very high speeds Ackermann’s steering model does not compensate for the large difference in slip angle between the inner and outer front tyres, an aspect that could cause some tracking imprecisions. So, when this speed situation is detected, according to Reference [53], a solution consists in using an Anti Ackermann geometry instead.

We use an EKF in combination with the Ackermann’s steering model to describe the motion of the vehicles. The EKF is a recursive Bayesian filter which iteratively repeats two steps at each frame: first, it estimates the object state ${\bar{x}}_{t}$ by applying the dynamic model to the previous state ${\bar{x}}_{t-1}$; second, it updates the estimated state ${\bar{x}}_{t}$ to the corrected state ${\bar{x}}_{t}$ for the current frame by fusing it with the new observation ${\bar{d}}_{t}$.

According to the dynamic model proposed, we define the following state transition function $f:R^{6}\to R^{6}$ as

(3) $${\bar{x}}_{t}=f\left({\bar{x}}_{t-1}\right)=\left[\begin{array}{c} x_{t-1}+v_{t-1}cos\left(\theta_{t-1}\right)∆t+\frac{1}{2}a_{t-1}cos\left(\theta_{t-1}\right)∆t^{2}\\ y_{t-1}+v_{t-1}sin\left(\theta_{t-1}\right)∆t+\frac{1}{2}a_{t-1}sin\left(\theta_{t-1}\right)∆t^{2}\\ \theta_{t-1}+\frac{1}{L}v_{t-1}tan\left(\phi_{t-1}\right)∆t\\ v_{t-1}+a_{t-1}∆t\\ \phi_{k-1}\\ a_{t-1} \end{array}\right]\;.$$

Note that *L* is the distance between the axles of the car, we fixed it to the value of 3.2 m in all our experiments. Note that the two so-called *driving processes*, $\phi_{t}$ and $a_{t}$, cannot be directly determined. They can be indirectly estimated by the EKF taking into account the remaining parameters when computing the Kalman gain.

**Figure 4:**
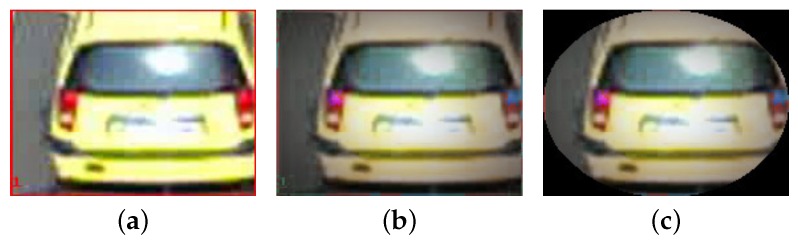
Pre-processing of detection window before computing its histogram. (**a**) Original image. (**b**) Image weighted with a Gaussian kernel. (**c**) Image masked with an ellipse. Reprinted by permission from Springer Nature: Springer, Natural and Artificial Computation in Engineering and Medical Application, (Vehicle Tracking by Simultaneous Detection and Viewpoint Estimation, Guerrero-Gomez-Olmedo, R. et al., 2013).

In this work we propose a hypothesize-and-verify framework for the tracker. Each vehicle trajectory hypothesis is defined as $H^{\left(i\right)}=\left[D^{\left(i\right)},A^{\left(i\right)}\right]$, where $D^{\left(i\right)}$ denotes its supporting detections $D^{\left(i\right)}=\left\{{\bar{d}}_{0}^{\left(i\right)},{\bar{d}}_{1}^{\left(i\right)},\ldots ,{\bar{d}}_{t}^{\left(i\right)}\right\}$ and $A^{\left(i\right)}$ is the appearance model encoding the appearance of the last instant of time. For $A^{\left(i\right)}$, we choose an (8×8×8)-bin color histogram in HSV space. As in Reference [41], given a bounding box via the detector, we do not directly compute the histogram for all its pixels. Instead, we pre-process the image within this detection window. It is extremely important to reject the portion of the bounding box that is not par of the vehicle, in order to be able to perform a good matching from one frame to another. Additionally, we must be resistant to small color variations produced by illumination changes. In order to accentuate the pixels located at the center of the detection, we process the image inside each bounding box using a Gaussian kernel to weight each pixel at position (x,y) as follows,

(4) $$\alpha_{x,y}=e^{\frac{{\left(x-x_{c}\right)}^{2}}{{\left(\frac{w}{\delta }\right)}^{2}}+\frac{{\left(y-y_{c}\right)}^{2}}{{\left(\frac{h}{\delta }\right)}^{2}}}\;,$$

where *w* and *h* are, respectively, the width and the height of the bounding box where the histogram is computed. $x_{c}$ and $y_{c}$ encode the center of the bounding box and δ is an empirical constant with a value of 2. Finally, we compute the histogram in HSV color space only for those weighted pixels inside an ellipse fitted to the bounding box, which is encoded in $a_{t}\left(i\right)$. This final step allows to ignore portions of the image located at the corners, that are typically asphalt, for instance. Figure 4 shows how this pre-processing steps work.

In each iteration, a new observation for vehicle *i*, that is, ${\bar{d}}_{t}^{\left(i\right)}$, is incorporated to the set $D^{\left(i\right)}$. The appearance model $A^{\left(i\right)}$ is updated as follows. If there is a new observation $a_{t}^{\left(i\right)}$, $A^{\left(i\right)}=a_{t}^{\left(i\right)}$. If there is no observation, the appearance model is updated using the last estimation $b_{t}^{\left(i\right)}$ given by the EKF but only if its appearance similarity with $A^{\left(i\right)}$ is under a threshold γ (we used γ= 0.2 in our experiments), otherwise $A^{\left(i\right)}$ is not updated, as it is gathered in the following equation:

(5) $$A^{\left(i\right)}=\left\{\begin{array}{cc} a_{t}^{\left(i\right)} & \mathrm{if}\;\mathrm{Card}(d_{t}^{\left(i\right)})=1\\ b_{t}^{\left(i\right)} & \mathrm{if}\;\mathrm{Card}(d_{t}^{\left(i\right)})=0\;\mathrm{and}\;\\ \; & d(b_{t}^{\left(i\right)},A^{\left(i\right)})<\gamma \\ A^{\left(i\right)} & \mathrm{otherwise}, \end{array}\right.$$

where Card$\left(d_{t}^{\left(i\right)}\right)$ means the cardinality of the set $d_{t}^{\left(i\right)}$ and d(·,·) is the Bhattacharyya distance that compares the appearance similarity of two appearance models as follows,

(6) $$d\left(b_{t}^{\left(i\right)},A^{\left(i\right)}\right)=\sqrt{1-\sum_{j}\frac{b_{t}^{\left(i\right)}\left(j\right)\cdot A^{\left(i\right)}\left(j\right)}{\sqrt{\sum_{j}b_{t}^{\left(i\right)}\left(j\right)\cdot \sum_{j}A^{\left(i\right)}\left(j\right)}}}\;.$$

This way of proceeding allows us to avoid incorporating asphalt information into the vehicle appearance model.

#### Data Association Algorithm

Matching observations with tracks is one of the most critical parts in our tracking algorithm. Some of the difficulties that have to be overcome by our approach are: missing measurements, track initiation problems, false alarms, multiple targets, crossing trajectories, etc. Furthermore, given the conditions and requirements we have (i.e., fast processing), we need an efficient algorithm.

**Figure 5:**
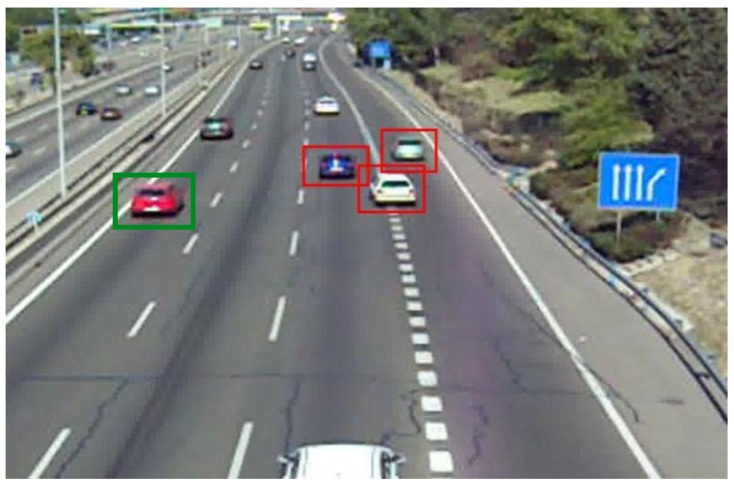
When assigning a new observation to a vehicle, that is, the matching process, it is better if we think locally, analyzing the surroundings of a vehicle, rather than thinking globally, trying to minimize a global cost function. In this case, the observations associated to the group of cars at the right should not be regarded when analyzing the red car at the left.

With this aim, we introduce an fast algorithm which employs both neighborhood and appearance models (See Algorithm 1). Essentially, our data association solution follows a zero-scan based approach, where only one hypothesis is allowed to remain after each iteration. Neighborhood and appearance models (Steps 1 and 2 in Algorithm 1) are introduced because the matching process is highly locally-dependent. For instance, see Figure 5. Observations related to the red car at the left in Figure 5 should be completely independent from the observations associated to the group of cars located at the right. However, for this second group, the assignments have to be done carefully and mostly based on the appearance and texture of the vehicles.

Overall, in our matching approach we proceed to incorporate the next three criteria into our algorithm:

1. Proximity (Algorithm 1—Step 1).
2. Appearance similarity (Algorithm 1—Step 2—Lines 1–22).
3. Detection overlap (Algorithm 1—Step 2—Lines 24–27).

Proximity criteria is developed in the first step of our algorithm. Technically, it consists in establishing a *dynamic neighborhood* for each tracked vehicle, which is defined by the overlap of the bounding boxes of the prediction of each track, $b_{t}^{\left(i\right)}$, with the new observations. That is, if there is no bounding box overlap, the vehicles involved are not allowed to interact during the data association. This is technically done by the function overlap in Algorithm 1, where we follow the standard overlap criteria proposed for the Average Precision metric of the official PASCAL VOC challenges [54] (see Equation (9)).

   **Algorithm 1** Data Association algorithm.
  **Inputs:** trackingList → H
  detectionList $\to {\bar{d}}_{t}$
  **Output:**
  trackingList (updated with the new observations) →H
 
  **Step 1:** *Establishing dynamic neighborhood*
 
 1 #Establishing dynamic neighborhood
 2 **for** each track **in** trackingList
 3   **for** each detection **in** detectionList
 4    **if** overlap(detection, track) #Overlap is measured using the corresponding bounding boxes
 5      add detection to track.neighborhood #Detection is added to the track list

 

  **Step 2:** *Assigning detections and disambiguation*
 
 1 **for** each track **in** trackinglist
 2   **if** size(track.neighborhood) = 0 #No vehicle selected
 3    track.currentStatus = NULL
 4 
 5   **else** **if** size(track.neighborhood) = 1 #Only one vehicle has been selected
 6    **if** appearanceSimilarity(track.neighborhood, #Checking the similarity in terms of appearance
 7          track.lastAppearance) < threshold
 8     track.currentStatus = track.neighborhood
 9    **else**
10     track.currentStatus = NULL
11 
12   **else** #More than one vehicle has been selected
13    **for** each neighbor **in** track.neighborhood
14     **if** appearanceSimilarity(neighbor, # Disambiguation by appearance similarity
15         track.lastAppearance) > threshold
16      delete neighbor
17 
18    **if** size(track.neighborhood) = 0
19     track.currentStatus = NULL
20 
21    **else** **if** size(track.neighborhood) = 1
22     track.currentStatus = track.neighborhood
23 
24    **else**  #Disambiguation by overlap criteria
25     neighbor = find_max_overlap(track.prediction,
26           track.neighborhood)
27     track.currentStatus = neighbor
 
  **Step 3:** *Avoiding more than one track sharing the same detection*
 
 1 **for** each detection **in** detectionList
 2   used = 0
 3   **list**.clean
 4 
 5   for each track **in** trackingList
 6    **if** track.currentStatus = detection
 7     used ++
 8     add track to **list**
 9 
10   **if** used > 1 #If it is used by more than one track ...
11    selected_track = find_max_overlap(detection, **list**) #…it select the one with maximum overlap
12 
13    **for** each vehicle **in** **list**
14     **if** vehicle **not** selected_track
15      vehicle.currentStatus = NULL
 
  **Step 4:** *Dealing with tracks without detections*
 
 1 **for** each track **in** trackinglist
 2   **if** track.currentStatus = NULL #Can we associate this empty track to a previous track?
 3    **if** appearanceSimilarity(track.prediction, #We check this situation using the appearance criterion
 4          track.lastAppearance) < threshold2
 5      track.currentStatus = track.prediction
 6 
 7    **else**
 8     remove track **from** trackingList #the vehicle is lost

 



After this step, it may happen that one vehicle $v^{\left(i\right)}$ has been assigned to zero, one or multiple neighboring observations $no_{t}^{\left(i\right)}$. In case no observations are assigned, i.e., Card $(no_{t}^{\left(i\right)})=0$, its supporting detection for that instant becomes empty, ${\bar{d}}_{t}^{\left(i\right)}=\emptyset$, and the prediction of the EKF for that instant of time, $b_{t}^{\left(i\right)}$, is used as a new *observation*, allowing us to continue tracking vehicle $v^{\left(i\right)}$ when it has been partially occluded or missed by the detector, see lines 1–11 of Step 2 in Algorithm 1. Although this assumption could be considered risky, our second criterion, i.e., Appearance similarity, corrects the situation in which the trajectory predicted by the filter is not satisfactory, removing the tracked vehicle from the list of vehicles occurring in the scene as described in Step 4 of Algorithm 1. The fact is that as long as we continue making predictions that are not backed by observations, the filter behavior tends to degrade. This degradation is not only dependent on how many times we use a prediction as an observation, but it is also related to the length of $D^{\left(i\right)}$, i.e., how much time we have been tracking vehicle $v^{\left(i\right)}$. A *long*$D^{\left(i\right)}$ means that we have incorporated many observations to its dynamic model, hence we have a good knowledge of its movement. Another factor is how much time the vehicle keeps loyal to the model obtained, and avoids sudden changes of speed or direction.

If only one observation is assigned, i.e., Card$(no_{t}^{\left(i\right)})=1$, our approach proceeds to associate the vehicle to the new observation if and only if the distance in Equation (6) is under a fixed threshold of 0.5, making ${\bar{d}}_{t}^{\left(i\right)}=no_{t}^{\left(i\right)}$. This is technically done by the function appearanceSimilarity in Algorithm 1.

If multiple candidate observations are assigned, i.e., Card$(no_{t}^{\left(i\right)})>1$, a different disambiguation criterion is needed. Let us assume we have *M* different candidates,

(7) $$no_{t}^{\left(i\right)}=\left\{no_{t}^{\left(i\right)}\left(1\right),no_{t}^{\left(i\right)}\left(2\right),\ldots ,no_{t}^{\left(i\right)}\left(M\right)\right\}\;.$$

We start selecting from $no_{t}^{\left(i\right)}$ those observations whose appearance similarity with $A^{\left(i\right)}$, using Equation (6), is under a threshold of 0.5 (See lines 5 – 11 of Step 2 in Algorithm 1). We call this set, ${\hat{no}}_{t}^{\left(i\right)}$. If Card$({\hat{no}}_{t}^{\left(i\right)})>1$, we need to use our third criterion, that is, Detection overlap, to select only one candidate as follows.

The higher the number of measurements, that we have incorporated into the model, Card($D^{\left(i\right)}$), the higher the confidence of our prediction. Therefore, making a good prediction of where the vehicle will appear in the next frame is more likely than making a bad prediction. Because of this, we proceed to assign to ${\bar{d}}_{t}^{\left(i\right)}$ the candidate in ${\hat{no}}_{t}^{\left(i\right)}$ reporting the biggest overlap with the current prediction of the EKF $b_{t}^{\left(i\right)}$,

(8) $${\bar{d}}_{t}^{\left(i\right)}=\underset{{\hat{no}}_{t}^{\left(i\right)}\left(m\right)}{arg max}\;\mathrm{ov}\left(b_{t}^{\left(i\right)},{\hat{no}}_{t}^{\left(i\right)}\left(m\right)\right)\;,$$

being ov(·,·) a function which measures the overlap between the bounding boxes. This is technically done by the function find_max_overlap in Algorithm 1, in which we use again the function overlap described above.

Overall, in Algorithm 1 the reader can see an step by step description with pseudo-code where we detail the proposed algorithm.

## 4. GRAM-RTM Database

**Figure 6:**
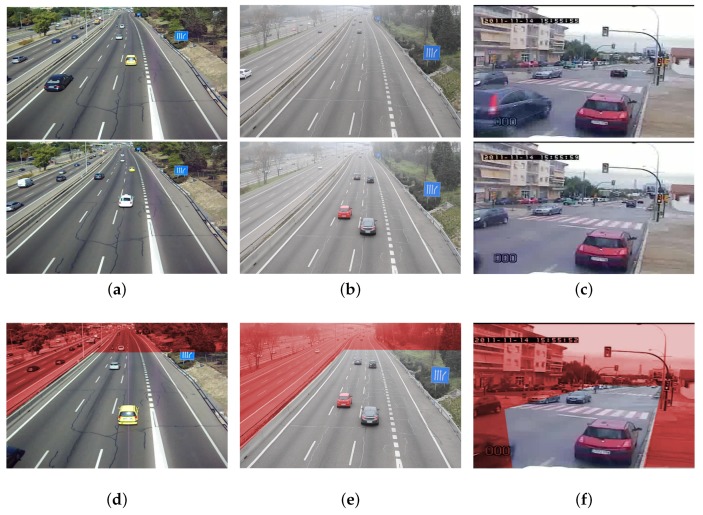
GRAM Road-Traffic Monitoring dataset images. Examples of images for the sequences: (**a**) M-30, (**b**) M-30-HD, (**c**) Urban1. The exclusion areas (in red) are shown in images (**d**), (**e**) and (**f**) for the M-30, the M-30-HD and the Urban1 sequences, respectively. Vehicles within these areas have not been annotated. Reprinted by permission from Springer Nature: Springer, Natural and Artificial Computation in Engineering and Medical Application, (Vehicle Tracking by Simultaneous Detection and Viewpoint Estimation, Guerrero-Gomez-Olmedo, R. et al., 2013.)

In this section we introduce the *GRAM Road-Traffic Monitoring* (GRAM-RTM) dataset, a benchmark for multi-vehicle tracking. It consists of 3 video sequences, recorded under different conditions and with different cameras. The first video, called M-30 (7520 frames), has been recorded in a sunny day with a Nikon Coolpix L20 camera, with a resolution of 800×480 @30 fps (frames per second). The second sequence, called M-30-HD (9390 frames), has been recorded in a similar location but during a cloudy day and with a high resolution camera: a Nikon DX3100 at 1200×720 @30 fps. The third video sequence, called Urban1 (23435 frames), has been recorded in a busy urban intersection with a video surveillance traffic camera with a resolution of 600×360 @25fps. Figure 6a–c show some examples of the images provided in the dataset.

All the vehicles in the dataset have been manually annotated using the tool described in Reference [55]. The following categories are provided: car, truck, van and big-truck. The total number of *different* objects in each sequence is: 256 for M-30, 235 for M-30-HD and 237 for Urban1. Note that only the vehicles that appear outside the red areas shown in Figure 6d–f have been annotated. A unique identifier for each annotated vehicle is provided. All these characteristics are summarized in Table 2. All the annotations included in the GRAM-RTM are created in a XML format PASCAL VOC compatible [54].

We publicly release the GRAM-RTM dataset (http://agamenon.tsc.uah.es/Personales/rlopez/data/rtm/), including the images, all the annotations and a set of tools for accessing and managing the database. Our aim is to establish a new benchmark for multi-vehicle tracking and detection for road-traffic monitoring applications using vision based sensors. For doing so, we establish the two following challenges with their corresponding experimental setups: vehicle detection and vehicle tracking.

### 4.1. Vehicle Detection

We start defining the task of vehicle detection in the GRAM-RTM dataset. For any of the vehicle classes provided, the objective is to predict the bounding boxes of each object of that class in a test image (if any). For every detection, a real-valued confidence associated to each bounding box should be provided, so that a precision versus recall curve can be obtained. For the evaluation metric, we propose to use the Average Precision (AP), which is the standard metric used in the object detection competition of the last PASCAL VOC challenges [54]. Recall that any detection in the red areas is discarded before the evaluation (We provide the tools to adequately analyze the results only considering the regions of interest defined.). So, for each detection defined by a bounding box $BB_{D}$, we measure its overlap with the ground truth bounding box provided $BB_{GT}$. A detection is considered valid if the computed intersection is over a threshold $\tau_{d}$ using the following formula,

(9) $$\frac{area(BB_{D}\cap BB_{GT})}{area(BB_{D}\cup BB_{GT})}>\tau_{d}\;,$$

where $\tau_{d}=0.5$.

### 4.2. Vehicle Tracking

For this second task, the objective is to track the vehicles in the three scenes. For the evaluation of the tracking, inspired by Reference [56], we propose to use the AP and precision versus recall curves again. That is, for each estimated bounding box given by the tracker $BB_{T}$, we measure its overlap with the ground truth bounding box provided $BB_{GT}$. An estimation is considered valid if it is over a threshold $\tau_{t}$ as follows,

(10) $$\frac{area(BB_{T}\cap BB_{GT})}{area(BB_{T}\cup BB_{GT})}>\tau_{t}\;.$$

Instead of using a fixed threshold $\tau_{t}$, we propose to compare the behavior of the different methods for different values of this overlap criterion. So, for the vehicle tracking problem, we propose to report the AP and the corresponding precision versus recall curve for $\tau_{t}=\left\{0.2,0.3,0.4,0.5\right\}$. This set of thresholds lets us analyze the tracking intrinsic behavior, where the lower the threshold, the less restrictive the evaluation in terms of object localization estimations. A similar strategy has been also considered within the context of object detection in Reference [57].

**Figure 7:**
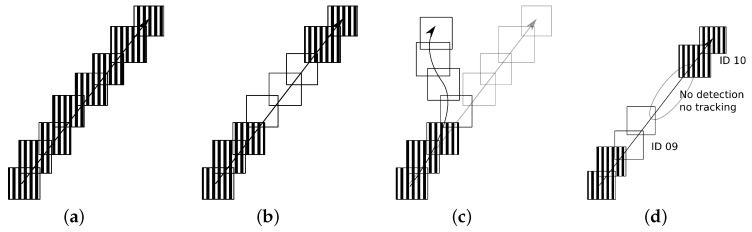
Tracking evaluation cases: (**a**) Detector and Tracker are correct. (**b**) Detector fails and Tracker is correct. (**c**) Detector and Tracker fail (only one vehicle is counted). (**d**) Detector and Tracker fail (more than one vehicle is counted). Reprinted by permission from Springer Nature: Springer, Natural and Artificial Computation in Engineering and Medical Application, (Vehicle Tracking by Simultaneous Detection and Viewpoint Estimation, Guerrero-Gomez-Olmedo, R. et al., 2013.)

With the proposed evaluation metric, the experimental validation considers the four cases shown in Figure 7. Note that the AP is adequate for measuring the four situations proposed. However, for penalizing the situation drawn in Figure 7d, we propose to also compare the different methods by simply providing the number of vehicles counted for each of the video sequences.

Finally, and in order to also evaluate the speed of the algorithms, we propose to report the frames per second (fps) rate, computed for the three video sequences in a total of 40,345 frames (7520 + 9390 + 23,435).

In summary, we do believe that with these four evaluations metrics (AP for detection, AP for tracking, number of vehicles counted and fps) GRAM-RTM can become a rigorous benchmark for establishing fair comparisons between different methods.

### 4.3. Best Practice: Recommendations on Using The Dataset

Please, note that the GRAM-RTM images must be used *never* for training, only for testing. Therefore, we propose the following experimental setup. Any approach reporting results in the GRAM-RTM database has to be trained using any data *except* the provided test data. Furthermore, the test data must be used strictly for reporting of results alone, that is, it must not be used in any way to train or tune systems, for example by running multiple parameter choices and reporting the best results obtained. For training, we suggest the use of other datasets providing vehicles, for example, PASCAL VOC [54].

## 5. Results

In this section, we present the experimental validation of our approaches in the GRAM-RTM dataset.

### 5.1. Technical Details

As it has been described in Section 3.1, we include in this experimental evaluation the two sensing solutions capable of performing a simultaneous object detection and viewpoint estimation. We call them HOG-Pose and FRCNN-Pose.

As a baseline, we include the experimental evaluation the mDPM model [19], which is also able to perform a simultaneous object detection and viewpoint estimation. Technically, we follow the original implementation (Code can be obtained from: http://agamenon.tsc.uah.es/Personales/rlopez/data/pose-estimation/) and train and test the mDPM for 4, 8 and 16 discrete poses.

HOG-Pose has been trained using the PASCAL VOC 2007 dataset [54]. For the pose, we only consider the four discrete poses annotated in that dataset, that is, frontal, rear, left and right. We use HOG features [10] which are parameterized with different values for the window and descriptor size, depending on the sequence to process. See Table 3 and Table 4 for all the details we used in our experiments. The use of different feature parameters depending on the sequence is motivated by the need of achieving a high detection rate.

FRCNN-Pose has been implemented using the deep learning framework Caffe [58]. The optimization is done by using the Stochastic Gradient Descent algorithm, with: a momentum of 0.9; a weight decay of 0.0005; and a learning rate of 0.001. The learning rate of the output layer for the pose estimation has been multiplied by a factor of 0.01, so as to guarantee that the network properly converges. We follow the standard procedure of the Faster R-CNN [16] for training the model in an end-to-end fashion. This way, for each training iteration, just one image is taken and passed through the first set of convolutions. In a second step, a collection of 128 region proposals is generated. These regions are used to build the batch to feed the last set of FC layers. This batch contains 32 samples of foreground samples and 96 samples of background. The dataset used for learning the FRCNN-Pose model is the publicly available PASCAL3D+ [59].

Note that before processing the images for the sequences M-30 and Urban, they have been scaled to the size of the M-30-HD, so all our images have a resolution of 1200×720.

### 5.2. Results

#### 5.2.1. Object Detection Results

First, we are going to analyze the results for the vehicle detection task, in order to provide a baseline to establish further comparisons with other detection methods.

**Figure 8:**
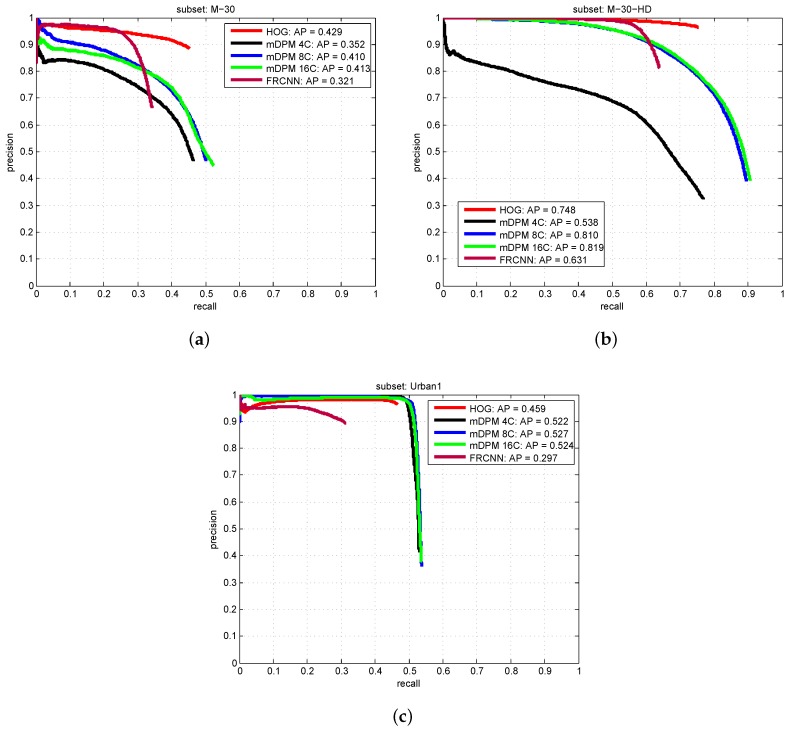
Precision vs. Recall curves for object detection: (**a**) M-30, (**b**) M-30-HD (**c**) Urban1.

Figure 8 shows the precision versus recall curves reported by all the approaches. These results reveal how challenging the GRAM-RTM is, where a recall higher than 60% has been only achieved in the M-30-HD sequence. HOG and mDPM-16 seem to report, on average, the best performance for all the models. However, in terms of speed, HOG is the clear winner, becoming the best option for the problem of interest. The main problem of the FRCNN solution is its low recall, although it reports a high and constant precision.

Within the context of surveillance applications, it is fundamental to control the number of false positives. The Urban1 sequence is specially challenging due to: the variation in viewpoint of the vehicles; the multiple occlusions and truncations of the objects present in the scene; and the low quality of the images. Overall, it seems that our detectors are able to deal with very different image resolutions and qualities.

The results reported in this paper outperform our preliminary approach in Reference [28]. We use now a more restrictive threshold for the detection (in Reference [28] $\tau_{d}$ was 0.2) and the HOG based approach obtains better AP for the three sequences. The increment of the performance with respect to Reference [28] resides also in a better training procedure with the PASCAL VOC 2007 images, which has been downscaled to obtain HOG templates which let us detect vehicles which appear far from the camera in the three sequences. Moreover, in this novel evaluation we report also the performance of the mDPM and FRCNN models.

#### 5.2.2. Vehicle Tracking Results

We now evaluate the tracking precision of the proposed approaches. As a baseline, we also report the results of a simple EKF, with the same dynamic model but where the pose of the object is *not* recovered through the detector. We call the models in this second type of approach as Model-NP (No Pose).

**Figure 9:**
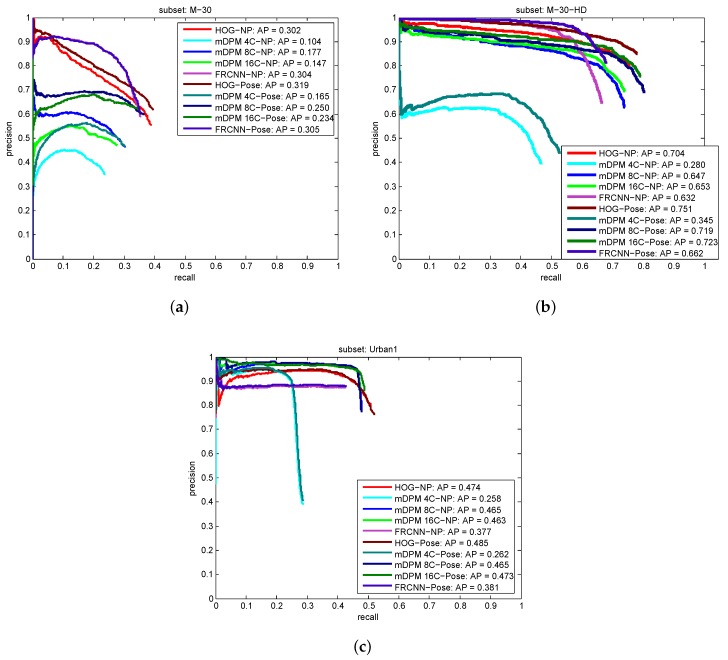
Precision vs. Recall curves for vehicle tracking for $\tau_{t}=0.5$: (**a**) M-30, (**b**) M-30-HD (**c**) Urban1.

**Figure 10:**
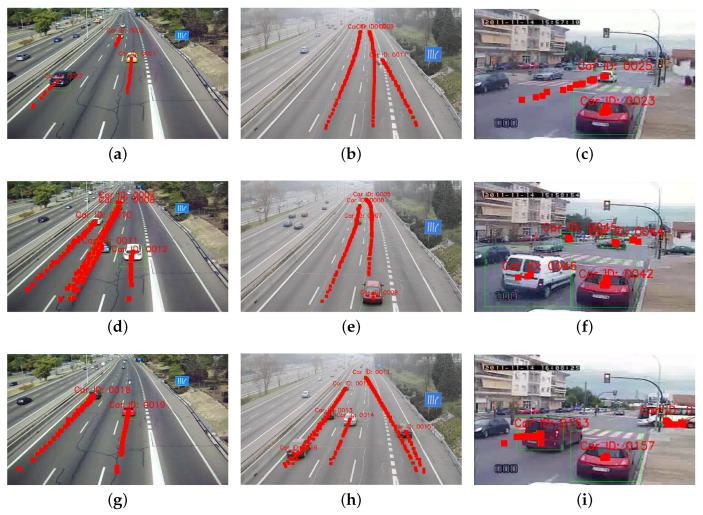
Qualitative results for the HOG-Pose model in M-30 (**a**,**d**,**g**), M-30-HD (**b**,**e**,**h**) and Urban1 (**c**,**f**,**i**).

Figure 9 shows the results reported by all the models and their variants. In general, when the pose is integrated in the EKF, the different approaches obtain the best performance, which corroborates the main hypothesis of our study: enhancing the tracking with observations of the vehicle pose, results in a better estimation of the vehicle motion. This increment is very relevant for the M-30-HD sequence, where the high quality of the images allows the detector to better estimate the viewpoints for the detections. These results also confirm that the robustness of the tracker relies on the video quality due to the data association step. The lower the resolution of the images, the more similar the histograms of close regions and the higher the number of incorrect matchings. Figure 10 shows qualitative tracking results using our HOG-Pose approach.

**Figure 11:**
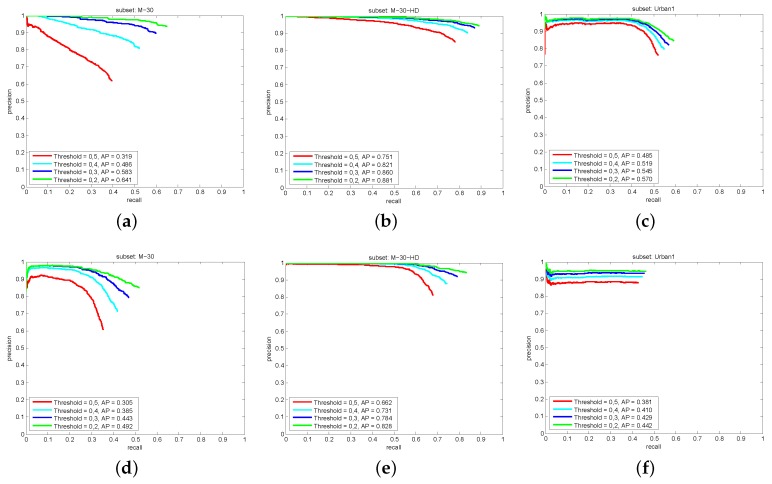
Tracking results for HOG-Pose in (**a**) M-30, (**b**) M-30-HD and (**c**) Urban1 and for FRCNN-Pose in (**d**) M-30, (**e**) M-30-HD and (**f**) Urban1. Note that different thresholds $\tau_{t}$ are used.

As it was described in Section 4.2, the evaluation metric proposed for the tracking can be used considering different thresholds $\tau_{t}$. In Figure 9 we show the results only for $\tau_{t}=0.5$. Figure 11 shows the tracking result for different values of $\tau_{t}$. One can for instance observe in Figure 11a, that simply relaxing the overlap criterion from 0.5 to 0.4, the tracking AP drastically increases (from 0.31 to 0.48). It is straightforward to think on situations which explain these results. For instance, we have in the scenes cars moving away from the camera. They become smaller and smaller, hence more difficult to detect. Although the detector fails, sometimes the tracker is able to follow them. In this situation, the tracker keeps the BB size of the last detection received. When we evaluate the performance of the tracker with the AP in Equation (10), we measure the overlap between the estimated BB and the ground truth BB. Therefore, in this situation, the further away the car is from the camera, the higher the difference between the size of the estimated BB and the ground truth BB. This means that, the higher the threshold $\tau_{t}$, the lower the percentage of estimations which are able to fulfill the overlap criterion. We consider important to relax the overlap criterion in the tracking evaluation. We do not want to penalize those systems that are able to keep tracking the vehicles in absence of measures, even when the vehicle size becomes smaller.

**Figure 12:**
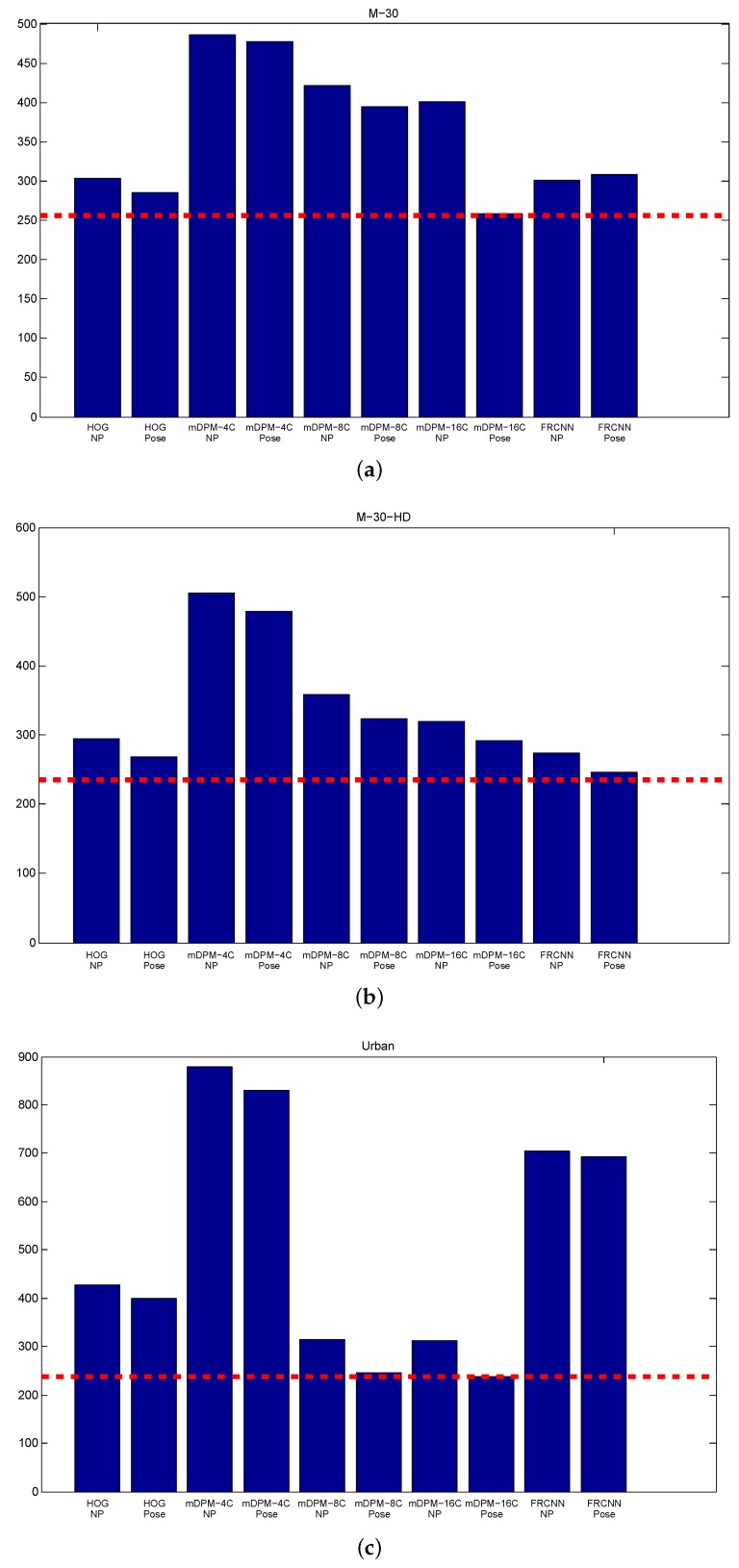
Counted Vehicles: (**a**) M-30, (**b**) M-30-HD (**c**) Urban1.

We also report the number of counted cars and the fps of all the approaches. First, Figure 12 shows that our EKF integrated with coarse viewpoint observations considerably reduces the error of counting cars with respect to an EKF without pose. The most challenging sequence is clearly Urban1. On average, both HOG-Pose and FRCNN-Pose get the best vehicle counting performance across the different scenes.

Finally, in terms of runtime, Table 5 offers a detailed comparative of different approaches for vehicle tracking and detection. If we observe the different image resolutions used, these results reveal that our solutions (both HOG and FRCNN) report competitive performances but, more importantly, both could be installed as solutions for a real vehicle counting system. The machine used for the analysis is an Intel Core 2 Quad @ 2.5 GHz machine, using a NVIDIA GeForce GTX 980 Ti GPU.

## 6. Conclusions

In this work our objective has been to present a boosting mechanism for multi-vehicle tracking, which consists in integrating in an EKF tracker a simultaneous object detection and viewpoint estimation vision based sensing solution. We have formulated the EKF in order to simultaneously integrate into the motion model both the position and the pose of the objects to be tracked. Two different models have been introduced for the simultaneous object detection and pose estimation problem: HOG-Pose and FRCNN-Pose. The thorough experimental evaluation carried in the challenging GRAM-RTM dataset confirms our hypothesis. We can conclude that enhancing a tracker by detection approach with a observations of the poses of the vehicles, results in a better estimation of the trajectories of the vehicles.

## Figures and Tables

**Table 1:** Comparison with state-of-the-art solutions for multi-vehicle tracking-by-detection solutions.

	Tracking-by-Detection	Integration of Pose Estimations	Pose Estimation Modifies the Motion Model
[6,41,42,43,44]	√	×	×
[47]	√	√	×
Ours	√	√	√

**Table 2:** GRAM-RTMdatabase details.

	M-30	M-30-HD	Urban1
Frames	7520	9390	23435
Resolution	800×480	1200×720	600×360
Vehicles annotated	256	235	237
Categories	car, truck, van, big-truck		

**Table 3:** Histogram of Oriented Gradients (HOG) settings (width, height) for M-30 and M-30-HD sequences.

	Frontal	Left	Rear	Right
HOG window	(54,39)	(50,19)	(54,39)	(50,19)
HOG descriptor	(6,4)	(7,3)	(6,4)	(7,3)

**Table 4:** HOG settings (width, height) for Urban1 sequence.

	Frontal	Left	Rear	Right
HOG window	(63,45)	(50,19)	(63,45)	(50,19)
HOG descriptor	(7,5)	(7,3)	(7,5)	(7,3)

**Table 5:** Time comparison of different vehicle tracking and detection approaches.

	fps	Resolution
HOG	11.13	1200×720
mDPM-4C	0.03	1200×720
mDPM-8C	0.015	1200×720
mDPM-16C	0.008	1200×720
FRCNN	1.488	1200×720
[41]	0.16	640×480
[47]	2.85	640×480

## Abbreviations

The following abbreviations are used in this manuscript:

KF	Kalman Filter
EKF	Extended Kalman Filter
HOG	Histogram of Oriented Gradients
DPM	Deformable Part Model
SVM	Support Vector Machine
MTT	Multi-Target Tracking
GNN	Global Nearest Neighbor
RPN	Region Proposal Network
MHT	Multiple Hypothesis Tracking
CNN	Convolutional Neural Network
R-CNN	Regions with CNN features
